# Identification, Antioxidant and Immunomodulatory Activities of a Neutral Exopolysaccharide from *Lactiplantibacillus plantarum* DMDL 9010

**DOI:** 10.3390/nu17142265

**Published:** 2025-07-09

**Authors:** Yanyan Huang, Weiting Liang, Yunhui Lu, Jie Xiong, Dongmei Liu, Xiangze Jia

**Affiliations:** 1 Guangdong Provincial Key Laboratory of Intelligent Food Manufacturing, College of Food Science and Engineering, Foshan University, Foshan 528225, China; huang_yanyan@fosu.edu.cn (Y.H.); 13431702370@163.com (W.L.); luyunhuiya@163.com (Y.L.); 2 School of Food Science and Engineering, South China University of Technology, Guangzhou 510640, China; jaydernxiong@163.com

**Keywords:** *Lactiplantibacillus plantarum* DMDL 9010, extracellular polysaccharide, structural characterization, antioxidant activity, immunoregulatory activity

## Abstract

Objectives: This study investigated the properties of a neutral exopolysaccharide (EPS-LP1) with an average molecular weight of 55,637 Da, isolated from *Lactiplantibacillus plantarum* DMDL 9010 (LP9010). Results: The composition of EPS-LP1 includes galactose (Gal), glucose (Glu) and mannose (Man) in a molar ratio of 5.35:86.25:8.40. Notably, EPS-LP1 exhibits a smooth and rod-like surface along with thermal stability. Methylation combined with nuclear magnetic resonance analysis revealed that EPS-LP1 structured as t-Gal*p*(1→, →6)-t Manp (1→)-Glc*p*(1→, 4)-Glc*p*(1→ and →4,6)-Gal*p*(1→), with relative molar ratio of 1.016:9.874:4.355:78.693:6.062, respectively. In the concentration range of 50 to 400 μg/mL, we observed the absence of cytotoxic effects from EPS-LP1 on RAW264.7 cells. Furthermore, EPS-LP1 demonstrated protective effects on RAW264.7 cells against oxidative damage by reducing the production of reactive oxygen species (ROS), malondialdehyde (MDA), and lactate dehydrogenase (LDH) release. Conversely, an increase in superoxide dismutase (SOD), catalase (CAT), glutathione peroxidase (GSH-Px), and concentrations of glutathione (GSH) was observed. Immunoreactivity assays indicated that EPS-LP1 can effectively reduce the production of nitric oxide (NO) and inhibit the secretion of tumor necrosis factor-α (TNF-α) and interleukin-6 (IL-6). Additionally, it inhibited the activation of the mitogen-activated protein kinase (MAPK)/nuclear factor-kappa B gene binding (NF-kB) signaling pathway. Conclusions: This research provides a foundation basis for further investigations into the neutral exopolysaccharide derived from LP9010.

## 1. Introduction

Probiotics refer to live microorganisms that provide advantageous physiological effects to host organisms when consumed in appropriate amounts, as defined by FAO/WHO (2006) [1]. Recent research has indicated that specific strains of *Lactobacillus*, particularly those within the genus *Lactobacillus*, can enhance both human and animal health [2]. *L. plantarum* is a gram-positive bacterium commonly found in fermented foods and the gastrointestinal tracts of animals. It plays an irreplaceable role in various sectors, including the food manufacturing sector, livestock nutrition manufacturing, and medical therapeutic applications [3].

Exopolysaccharides (EPS) represent intricate carbohydrate polymers composed of monosaccharide units originating from sugar compounds and their derivatives, generated by diverse organisms such as bacteria, fungi, algae, and plants [4]. Particularly noteworthy are EPS synthesized by food-safe lactic acid bacteria (LAB), which demonstrate potential as viable alternatives to traditional plant- and animal-derived EPS in various applications [5]. These biopolymers are primarily grouped into two categories: capsular polysaccharides, which form protective cellular coatings, and mucopolysaccharides with viscous properties, with additional subdivisions determined by their structural configurations and functional characteristics. Microorganisms synthesize extracellular polysaccharides (EPS) that can be categorized as homogeneous or heterogeneous polysaccharides. Current studies reveal that microbial-derived EPS possess a wide array of bioactive properties, including antitumor potential, immune regulation, hypoglycemic effects, blood-thinning characteristics, antiviral activity, and oxidative stress mitigation [2,6]. The structure of EPS plays a crucial role in determining its biological activity. *L. plantarum* is one of the predominant species known for producing EPS, but the structural characteristics of polysaccharides extracted from *L. plantarum* remain inadequately understood. Consequently, a comprehensive investigation into the physicochemical properties and biological activities of *L. plantarum*-derived EPS is essential to enhance its applications in functional foods as well as other industries.

Recent years have witnessed growing attention toward the medicinal potential and health-promoting properties of extracellular polysaccharides (EPS). Elevated oxidative stress, stemming from excessive accumulation of reactive oxygen species (ROS) and unstable free radical molecules within living organisms [7], plays a significant role in disease development processes. Dietary antioxidants play a crucial role in maintaining health by neutralizing harmful ROS and unstable radicals through an electronic delivery mechanism. Notably, naturally occurring polysaccharides exhibit promising capabilities in boosting cellular defense systems against oxidative damage while demonstrating therapeutic efficacy. These bioactive compounds demonstrate immunoregulatory potential through macrophage activation, emerging as viable candidates for immune system modulation [8,9]. As pivotal mediators of immune responses, macrophages orchestrate both innate and adaptive immunity by secreting multiple inflammatory mediators, including nitric oxide (NO), tumor necrosis factor-alpha (TNF-α), interleukin-1beta (IL-1β), and interleukin-6 (IL-6) following stimulation. These immunomodulators demonstrate potent inhibitory effects against pathogenic microorganisms and malignant cell proliferation [10,11]. However, given that the extracellular polysaccharide derived from *L. plantarum* exhibits antioxidant activity, it is necessary to explore its potential immunomodulatory effects further.

The objective of this study was to conduct a preliminary investigation into the physical properties of EPS produced by probiotic strain *L. plantarum* DMDL 9010 (LP9010). LP9010 exhibits notable resistance to acidic environments and gallstones, possesses cholesterol-lowering capabilities, and demonstrates significant nitrite-degrading ability [12,13,14]. This strain can serve as an effective starter culture in fermented dairy products. This study aimed to evaluate whether EPS derived from LP9010 possesses antioxidant and immune-modulating properties. To achieve this goal, the macrophage-induced oxidative stress model was treated with H_2_O_2,_ alongside a macrophage-induced immune damage model, which was stimulated by lipopolysaccharide (LPS). The results of this study will offer evidence regarding the structure–activity relationship between the intrinsic properties of EPS and their antioxidant and immune activity.

## 2. Materials and Methods

### 2.1. Strains and Exopolysaccharides Production

The experimental strain utilized in this study was *L. plantarum* DMDL 9010, which was isolated from traditional Chinese pickles in our laboratory and is currently preserved at the General Microbial Species Preservation and Management Center of China. The genome sequence entry numbers are CP063986–CP063988 [15]. The method for preparing EPS using LP9010 was based on previous studies. Initially, 1 × 10^9^ CFU/mL LP9010 was inoculated at a concentration of 2% (*v*/*v*) into modified sugar-free MRS (Qingdao Hope Bio-Technology Co., Ltd., Qingdao, China) medium and fermented at 37 °C for 18 h. The inoculated LP9010 samples were all activated twice. Following fermentation, the resulting liquid (1000 mL) was centrifuged at 8000× *g* and 4 °C for 15 min, after which the collected supernatant was concentrated using a rotary evaporator (RE-2000A, Shanghai Yarong, Shanghai, China).

### 2.2. Isolation and Purification of EPS

The separation and purification steps were conducted in accordance with a previous study [16], with minor modifications. Protein precipitation was performed at 4 °C for 24 h using the Sevag method (n-butanol and chloroform at a volume ratio of 1:4), resulting in the acquisition of crude EPS following deproteinization. The EPS solution was subsequently lyophilized for further analysis. The crude lyophilized EPS was reconstituted into a 20 mg/mL solution and centrifuged at 8000× *g* for 10 min. The supernatant was then filtered through a 0.22 μm filter membrane. To obtain pure polysaccharide, the crude polysaccharide underwent purification utilizing DEAE-Cellulose-52 anion exchange column (26 × 400 mm GE Healthcare, Beijing, China) and Sephadex G75 gel filtration column (16 × 500 mm GE Healthcare, Beijing, China). Details regarding the purification conditions can be found in Supplementary Table S1 and Figure S1. The eluent collected from each tube was measured, and the purified EPS-LP1 exhibiting the highest absorption peak was gathered and freeze-dried for subsequent experiments.

### 2.3. Scanning Electron Microscopy (SEM) Analysis

To investigate the microscopic morphology, a 10 nm thick gold layer was deposited onto the surface of EPS-LP1 (5 mg) following purification and drying. The surface morphology was subsequently examined using SEM (Akishima, Tokyo, Japan) at 20 kV [11].

### 2.4. Ultraviolet Spectral Analysis and Molecular Weight Analysis

The UV spectrum (Thermo Fisher Scientific, Waltham, MA, USA) was rapidly scanned within the wavelength range of 200~520 nm to obtain the UV absorption spectrum of EPS-LP1.

The molecular weight of the purified EPS-LP1 was determined using Huang’s method [17] via high-performance gel permeation chromatography (HPGPC) with a TSK-gel-G3000PWXL column (0.78 cm × 30 cm, Tokyo, Japan). Diverse dextran standards ranging from 5.0 to 670 kDa were utilized as references for calculating the molecular weight.

### 2.5. Thermogravimetric (TG), and FT-IR Spectroscopy

The thermal stability of EPS-LP1 (25 mg) was assessed using a TG analyzer (PerkinElmer, Waltham, MA, USA) in a nitrogen atmosphere. The analysis was conducted at a heating rate of 10 °C/min over the temperature range of 25–800 °C.

For FT-IR analysis, the EPS-LP1 sample was mixed with dry KBr, constituting 0.1% of the total sample weight. The mixture was then ground and pressed into pellets with a thickness of 1 mm. FT-IR spectra were obtained using an FT-IR spectrometer (Nicolet 6700, Thermo Fisher Scientific, Waltham, MA, USA), covering an infrared range from 500 to 4000 cm^−1^ to identify the presence of functional groups within the sample [18].

### 2.6. Analysis of Monosaccharide Composition

To determine the monosaccharide composition, a purified EPS-LP1 sample (5 mg) was hydrolyzed in 4 mL trifluoroacetic acid (TFA) at a concentration of 2 mol/L under conditions of 121 °C for a duration of 2 h. Following hydrolysis, the TFA in the sample was evaporated using nitrogen gas, and the residual acid was eliminated through decompression and methanol distillation, with this process being repeated three times [19,20]. The resulting samples were then dissolved in sterile water and filtered through a 0.22 µm filter before undergoing analysis via high-performance liquid chromatography (HPLC) (ICS5000+, Thermo Fisher Scientific, Waltham, MA, USA) with a Dionex™ CarboPac™ PA10 liquid chromatography column (250 4.0 mm, 10 m, Thermo Fisher Scientific, Waltham, MA, USA) [17]. Specific chromatographic conditions can be found in Supplementary Table S2. The standard monosaccharides employed for comparative analysis included fucose, arabinose, galactose, glucose, and xylose, among other monosaccharides.

### 2.7. Methylation Analysis and Nuclear Magnetic Resonance (NMR) Analysis

For methylation analysis, EPS-LP1 (10 mg) was dissolved in sterile water and subsequently reacted with carbodiimide (100 mg/mL, 1 mL) for a duration of 2 h. Following this, an imidazole solution (2 mol/L, 1 mL) was introduced into the reaction mixture, which was then divided into two parts. Sodium borohydride (NaBH_4_; 30 mg/mL, 1 mL) and sodium deuteride borohydride (NaBD_4_; 30 mg/mL, 1 mL) were added to each part, respectively, and allowed to react for another 3 h. The reaction was terminated by the addition of glacial acetic acid (100 μL). The resulting samples were dialyzed and lyophilized before being treated with dimethyl sulfoxide (500 μL) to perform methylation according to previous studies [21,22,23]. The methylation derivatives were analyzed using gas chromatography–mass spectrometry (GC–MS; model: 7890A-5977B, Agilent Technologies, Palo Alto, CA, USA), equipped with a quadrupole mass spectrometer system and a DB-5 ms fused silica capillary column (dimensions: 0.25 mm × 0.25 μm × 30 m). High-purity helium served as the carrier gas at a split ratio of 10:1. The initial temperature of the column incubator was set at 140 °C for a period of 2 min, followed by a temperature ramping program that reached up to 230 °C at a rate of 3 °C/min over the course of an additional three minutes.

The purified EPS-LP1 was subsequently analyzed using a nuclear magnetic resonance (NMR) spectrometer (Bruker Corporation, Karlsruhe, Germany) to obtain ^1^H and ^13^C spectra [24]. The NMR analysis method involved dissolving a 60 mg sample of the purified EPS in 0.6 mL of heavy water (D_2_O), stirring at room temperature for 2 h, and then freeze-drying the solution. This process was repeated three times. Finally, the sample was dissolved again in 0.6 mL D_2_O and transferred into an NMR tube for detection.

### 2.8. Cellular Antioxidant Activity

#### 2.8.1. Cell Culture and Cell Viability Assay

According to Zhou’s method [25], mouse macrophages (RAW264.7) were cultured in DMEM medium supplemented with 10% FBS and 1% streptomycin–penicillin at 37 °C in an incubator (MCO-18 AC, Panasonic, Osaka, Japan) under a controlled atmosphere of 5% CO_2_ and saturated humidity. The impact of EPS-LP1 on the growth of RAW264.7 cells was assessed using the MTT assay [26]. RAW264.7 cells were seeded into 96-well plates at a density of 1 × 10^6^ cells per well and maintained in an incubator with a CO_2_ concentration of 5% at 37 °C for 24 h. Following this incubation period, various concentrations (0, 50, 100, 200, and 400 μg/mL, each volume being 100 μL) of EPS-LP1 solutions were administered to the wells for an additional treatment period of two hours. Control groups included blank control cells treated with DMEM containing H_2_O_2_ at a concentration of 1 μmol/L and a positive control group receiving vitamin C (Vit C) at a concentration of 100 μg/mL. Subsequently, RAW264.7 cells were incubated in the presence of light-excluded conditions with MTT solution (10 μL per well) for two hours to detect cell viability. The absorbance values for each cell were measured at a wavelength of 570 nm using an enzyme-linked immunosorbent assay reader (1510, Thermo Fisher Scientific, Waltham, MA, USA).

#### 2.8.2. Intracellular Reactive Oxygen Species

Reactive oxygen species (ROS) in RAW264.7 cells were quantified using the DCFH-DA method, as previously described by Yingying Wang [27]. Initially, the cells were seeded into 96-well plates at a concentration of 1 × 10^6^ cells per well and treated with varying concentrations (0, 50, 100, 200, 400 μg/mL, 100 μL) for 2 h. Following this incubation period, the cells were exposed to 1 μmol/L H_2_O_2_ overnight for an additional 24 h and subsequently digested with trypsin (0.25%) at room temperature until they assumed a rounded morphology. The reaction was terminated by washing the cells with DMEM complete medium to remove any residual H_2_O_2_. The harvested cells were then transferred into centrifuge tubes, and subjected to centrifugation at 1000× *g* for five minutes at 4 °C; after this, the supernatant was removed. Subsequently, DCFH-DA (10 μmol/L) was added to each tube and incubated at 37 °C for 20 min, and after another round of centrifugation, the cells were washed with DMEM culture medium to eliminate any unbound DCFH-DA remaining in the system. Following this step, PBS washings were performed before analyzing intracellular levels of reactive oxygen species using FACS Calibur flow cytometry (FACS Calibur, BD, Franklin Lake, NJ, USA), all within one hour post-treatment. DMEM served as a blank control while Vit C (100 μg/mL) acted as a positive control in these experiments.

#### 2.8.3. Determination of Cellular Antioxidant Status

The experimental group received treatment with test compounds, while vitamin C (100 μg/mL) served as the positive control, and untreated cells acted as the negative control in these assays. For this investigation, cells were seeded into 96-well plates at a density of 1 × 10^6^ cells per well. After seeding, the cultures were treated with 100 μL of EPS-LP1 at different concentrations (0, 50, 100, 200, 400 μg/mL) for 2 h. Following treatment, cellular systems were challenged with 1 μmol/L hydrogen peroxide (H_2_O_2_) overnight (24 h). After the exposure period, samples were collected and centrifuged. The cellular secretion profiles of malondialdehyde (MDA), superoxide dismutase (SOD), glutathione (GSH), glutathione peroxidase (GSH-PX), catalase (CAT), and lactate dehydrogenase (LDH) were evaluated by applying the super serum following the methodology outlined in [28]. Vitamin C (100 μg/mL) was employed as the positive control, with DMEM serving as the blank control group.

### 2.9. Immunoregulatory Activity

#### 2.9.1. Cell Survival Analysis

The activity of RAW264.7 cells was evaluated using the CCK-8 assay. Specifically, RAW264.7 cells were inoculated in 96-well plates at a concentration of 1 × 10^6^ cells per well, with a total volume of 100 μL during the logarithmic growth phase. The culture was incubated overnight for 24 h in fresh DMEM medium at 37 °C in an incubator containing 5% CO_2_. In the experimental setup, DMEM medium served as the blank control, and LPS acted as the positive control. After discarding the supernatant, DMEM medium with varying concentrations (0, 50, 100, 200, 400 μg/mL, each with a volume of 100 μL) was added to each well, followed by another overnight incubation period lasting for an additional 24 h. Subsequently, a solution of CCK-8 (10% *v*/*v*) was introduced into each well and further cultured for an additional four hours. The absorbance at a wavelength of 450 nm was then measured using an enzyme-labeled plate reader (Thermo Fisher Scientific, Waltham, MA, USA).

#### 2.9.2. Nitric Oxide (NO) and Cytokines Analysis

RAW264.7 macrophages were seeded in 96-well culture plates at an initial density of 1 × 10^6^ cells/mL in a 96-well plate at 100 μL per well. The cellular cultures were subsequently administered with EPS-LP1 polysaccharide solutions prepared in different concentration gradients (0, 50, 100, 200, and 400 μg/mL). The experimental procedure involved administering treatments to cell cultures in a final volume adjusted to 100 μL for two hours. Post-treatment application, LPS stimulation was performed at 1 μg/mL concentration with subsequent 24 h incubation. Cellular supernatants were harvested through centrifugal separation (10,000 ×g, 10 min at 25 °C) and preserved at −20 °C prior to analysis. Quantitative measurement of nitric oxide levels in collected supernatants was conducted using a commercial detection kit (Nanjing, China) [29]. Parallel evaluations included cytokine profiling through interleukin-6 (IL-6) and tumor necrosis factor-alpha (TNF-α) concentration measurements. The concentrations of tumor necrosis factor-alpha (TNF-α) in supernatant samples were quantified using a commercially available ELISA detection kit (Jiancheng, Jiangsu, China), with experimental procedures performed following the guidelines supplied by the manufacturer. Normal DMEM medium without any stimulant was used as the blank control group.

#### 2.9.3. Western Blotting Analysis

Western blotting was performed to determine the expression levels of key phosphorylated proteins in MAPK and NF-κB signaling pathways using RAW264.7 cells. Specifically, the phosphorylated forms of protein-38 (p-P38), c-Jun N-terminal kinase (p-JNK), and extracellular signal-regulated kinases (p-ERK) were quantified in the MAPK pathway. Additionally, the activation status of NF-κB pathway components was examined through measurements of phosphorylated P65 (p-P65) and phosphorylated inhibitory subunit IκB-α (p-IκB-α) [30]. The relative protein levels were normalized to β-actin, which served as an internal control.

### 2.10. Statistical Analysis

Data analysis and plotting were performed using GraphPad Prism (version 8.0.1) and SPSS Statistics 26 (IBM, Armonk, NY, USA). Results were presented as mean ± standard deviation, with variance analysis conducted using the Tukey test *(p* < 0.05). One-way analysis of variance (ANOVA) was employed to evaluate differences among all groups. A significance level of *p* < 0.05 was considered significant when comparing results to those of the control group or the LPS-induced group.

## 3. Results

### 3.1. Production, Isolation, and Purification of Exopolysaccharide

**Figure 1:**
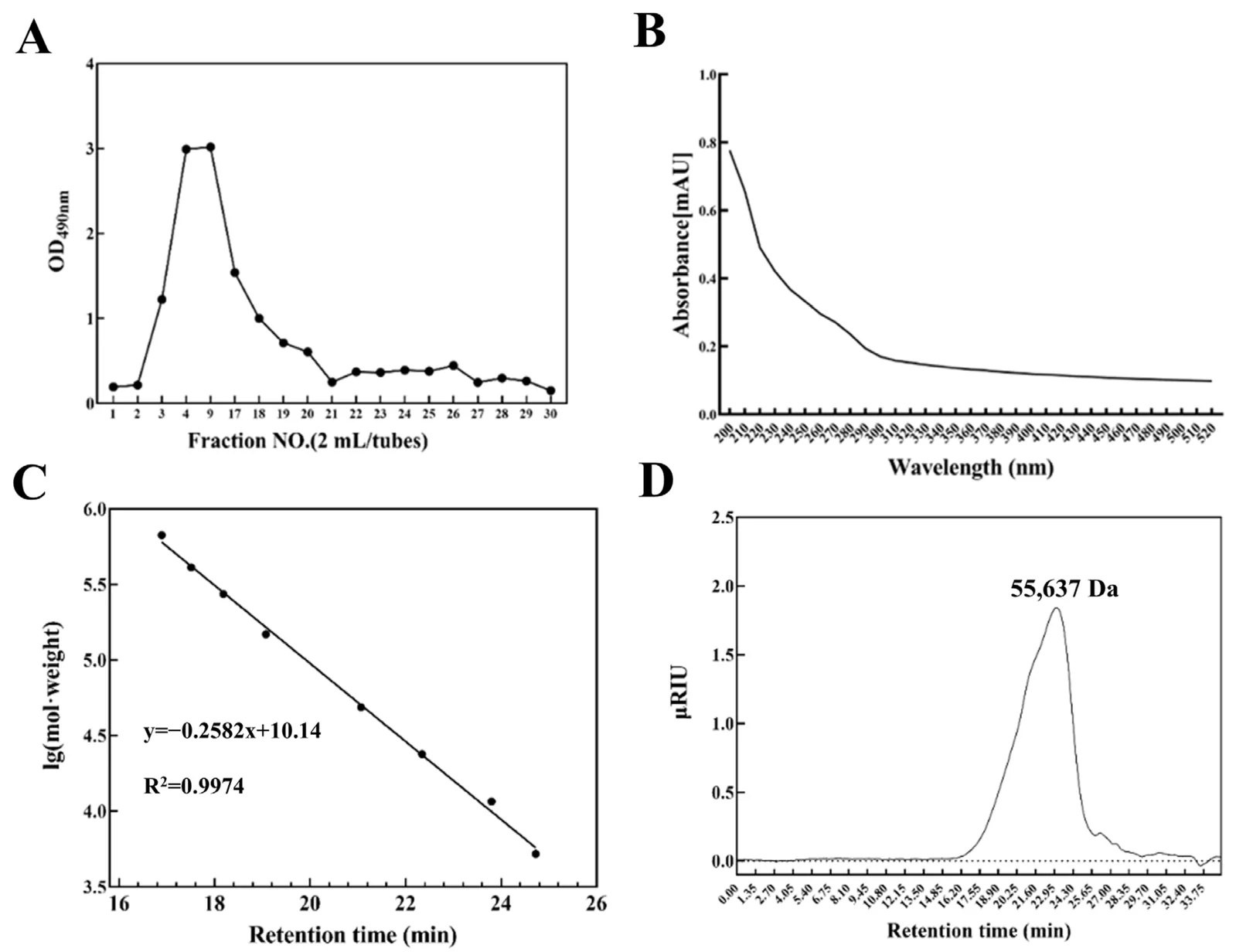
Production, separation, purification, and molecular weight of EPS from LP9010. (**A**) Chromatograms of EPS purified by Sephadex G-75 column, (**B**) UV, (**C**) standard curve of polysaccharide molecular weight (5.0–670 kDa), and (**D**) HPGPC spectrum of EPS-LP1.

The crude exopolysaccharide (EPS) of LP9010, with a yield of 0.5641 g, was obtained through deproteinization and lyophilization. To isolate single-component polysaccharides, further separation and purification were conducted using a DEAE-Cellulose-52 anion exchange column. EPS-LP1 exhibited the most pronounced absorption peak (Figure S1). EPS-LP2 has been demonstrated to possess excellent antioxidant activity and remarkable immunomodulatory activity, as shown in Figure S1 [17]. Components separated by the ion-exchange column often possess similar charge properties but may contain components with varying molecular weights. Consequently, a significant quantity of crude EPS-LP1 was collected and purified via Sephadex G-75 column to obtain products of uniform molecular weight (Figure 1A).

### 3.2. Ultraviolet Spectral Analysis and Molecular Weight Analysis

The purity evaluation of EPS-LP1 was conducted through UV–Vis spectrophotometric analysis. Distinct absorbance maxima at 260 nm and 280 nm in the ultraviolet spectrum are characteristic indicators of nucleic acids and protein contamination. Notably, the purified EPS-LP1 preparation demonstrated negligible absorbance at both critical wavelengths, suggesting the absence of contaminants and high purity levels (Figure 1B). This observation aligns with prior research conducted by the result of Wang et al. [31], where analogous purification techniques were implemented to achieve comparable purity standards in polysaccharide samples.

The molecular weight of EPS-LP1 was determined using HPGPC in conjunction with a standard curve based on dextran anhydride standards (y = −0.2582 x + 10.14, R^2^= 0.9974, Figure 1C). The calculated molecular weight of EPS-LP1 is approximately 55,637 Da. The chromatogram for the EPS-LP1 component displayed a symmetrical single peak (Figure 1D), indicating that EPS-LP1 is a homogeneous polysaccharide component.

### 3.3. Scanning Electron Microscopy (SEM) Analysis

**Figure 2:**
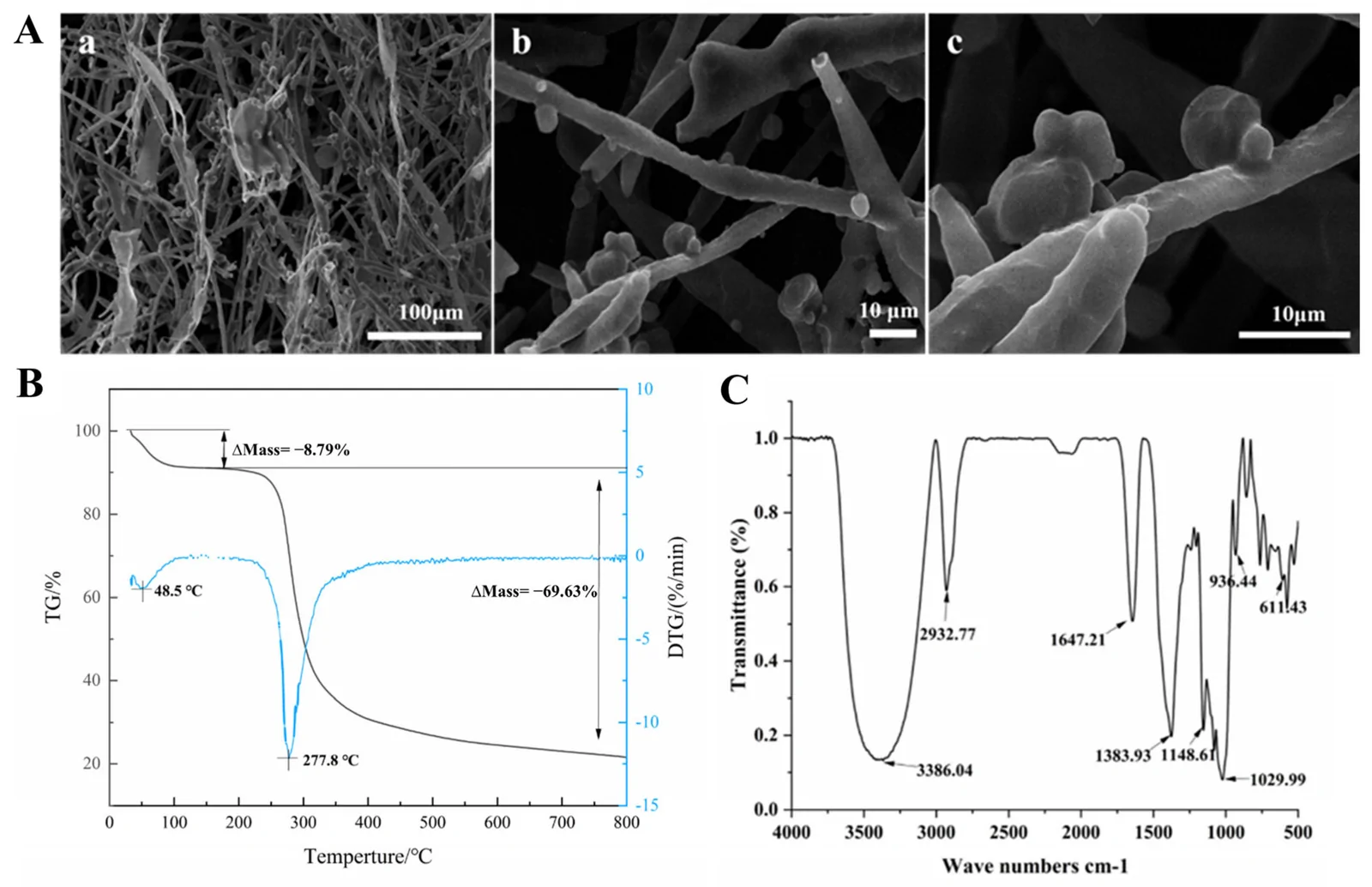
General properties analysis of EPS-LP1. (**A**) SEM (a) 100 μm, (b) 10 μm, and (c) 10 μm, (**B**) Thermogravimetric (TG) analysis curve of EPS-LP1, and (**C**) FT-IR spectra.

Under high magnification using SEM, the surface morphology of biomacromolecules can be visualized, allowing for detailed observation of polymer characteristics. This provides valuable insights into the properties of polymers and enhances our understanding of the physical attributes of EPS-LP1 [32]. The surface of EPS-LP1 exhibits a smooth and glossy appearance, characterized by a rod-like structure (Figure 2(Aa)). Furthermore, there is evidence of partial adhesion between the spherules within the EPS matrix and its branches (Figure 2(Ab,c)). The highly branched network structure of EPS-LP1 significantly contributes to enhancing various physical and chemical properties, including increased viscosity, improved texture, and enhanced water-holding capacity [33].

### 3.4. Thermogravimetric (TG) Analysis and FT-IR Spectroscopy

TG analysis technology was employed to investigate polysaccharides and their thermal stability characteristics of EPS. The results indicated that EPS-LP1 underwent degradation through two consecutive weight loss stages (Figure 2B). The initial weight loss, occurring before reaching 198.1 °C (8.79%), can be primarily attributed to the evaporation of water or the loss of other volatile substances. However, between 198.1 °C and 800 °C, EPS exhibited a significant weight loss (69.6%), indicating that EPS-LP1 demonstrates distinct thermal stability without any nutrient loss from its surface during heating. This property guarantees the integrity and dependability of EPS-LP1 when incorporated into cultured milk products, as no bioactive component depletion occurs even under elevated temperatures.

The main functional groups present in EPS-LP1 were analyzed using Fourier transform infrared (FT-IR) spectroscopy. Figure 2C shows a prominent absorption peak located at 3386.04 cm^−1,^ which arises from O-H stretching vibrations in the polysaccharide’s hydroxyl groups [34]. The spectral feature observed at 2932.77 cm^−1^ corresponds to asymmetric C-H stretching vibrations associated with the aliphatic CH_2_ groups [35]. Furthermore, the absorption band appearing at 1647.21 cm^−1^ exhibits spectral characteristics comparable to those documented for mannose and galactose residues [36], while the peak at 1383.93 cm^−1^ may result from symmetric stretching vibrations of the carboxyl groups [16]. The two strong absorptions at 1148.61 cm^−1^ and 1029.99 cm^−1^ suggest that EPS-LP1 may contain pyran residual sugar groups, corroborated by the peaks at 936.44 cm^−1^ and 611.43 cm^−1^, which may be asymmetric and symmetric stretching vibrations of the pyranose ring, respectively [35]. These absorption peaks observed in the range of 1200–1000 cm^−1^ suggest the presence of C-O-H and C-O-C tensile vibrations within EPS-LP1’s molecular structure. Furthermore, a prominent absorption band identified at 1029.99 cm^−1^ indicates an abundance of polysaccharides present in this sample [37].

### 3.5. Monosaccharide Composition, Methylation, and Nuclear Magnetic Resonance Analysis of EPS-LP1

**Figure 3:**
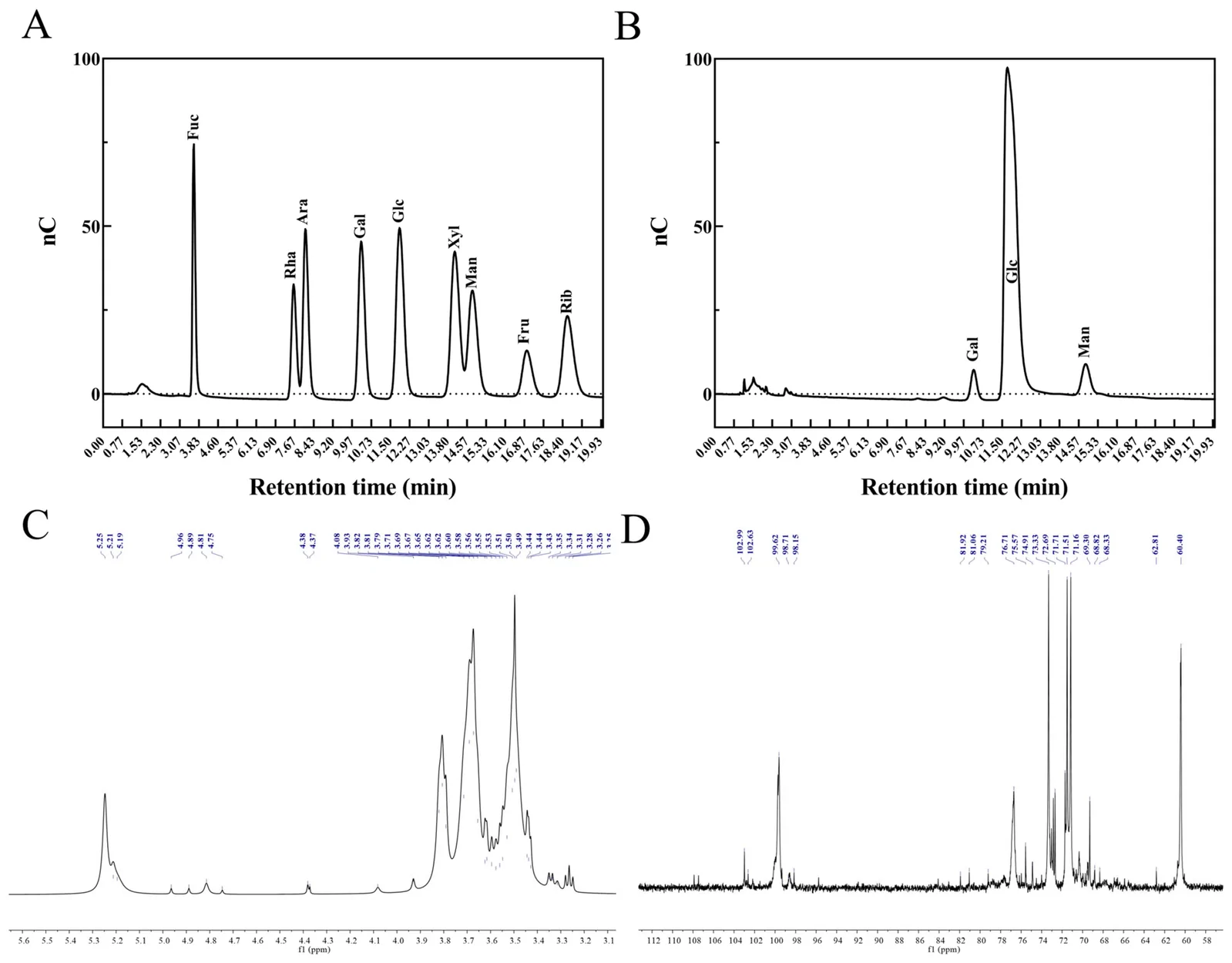
Monosaccharide composition analysis and NMR spectra of EPS-LP1. (**A**) HPLC analysis of monosaccharide composition of monosaccharide standard product (Fuc = fucose, Rha = rhamnose, Ara = arabinose, Gal = galactose, Glc = glucose, Xyl = xylose, Man = mannose, Fru = fructose, Rib = ribose); (**B**) HPLC analysis of monosaccharide composition of EPS-LP1; (**C**) ^1^H NMR spectrum; (**D**) ^13^C NMR spectrum.

The analysis of monosaccharide composition indicated that EPS-LP1 is a heteropolysaccharide comprised of various sugar monomers, including galactose (Gal), glucose (Glu), and mannose (Man), in a molar ratio of 5.35:86.25:8.40, with glucose being the most predominant component (Figure 3A,B).

GC-MS methylation analysis was conducted to elucidate the types of linkages between glycosylated residues (Supplementary Table S2). The glycosidic bonds in EPS-LP1 were characterized as follows: t-Gal*p*, t-Man*p*, 6-Glc*p*, 4-Glc*p*, and 4,6-Galp with relative molar ratios of 1.016, 9.874, 4.355, 78.693, and 6.062, respectively. The relative molar ratio of terminal units (t-Gal*p* and t-Man*p*) to branch points (4, 6-gal*p*) is calculated to be approximately 1.796, indicating that there are nearly twice as many terminal units as branch points, and implying that each branch may connect to two terminal units (t-Gal*p* and t-Man*p*). Using the equation DB = (NT + NB)/(NT + NB + NL), the branching degree (DB) of EPS-LP1 has been determined to be approximately 16.95%, where NT represents the terminal residual bases t-Gal*p*(1→) and t-Man*p* (1→); NB denotes the branching residue →4,6-Gal*p* (1→); while NL indicates the linear residues →6Glc*p*(1→ and 4)-Glc*p*(1→).

The ^1^H NMR spectra were employed to investigate the composition of the glycosidic bond in EPS-LP1 (Figure 3C). The ^1^H NMR spectrum contained signals for three anomeric protons at δ5.25, 5.21, and 5.19 ppm, indicating that EPS-LP1 was mainly composed of three types of sugars. Broad resonance peaks spanning δ3.4–4.0 ppm were observed, characteristic of oxygen-bound CH and CH2 groups within carbohydrate structures. These shifts corresponded to protons located at positions H-2 through H-6 in the sugar moieties. The distribution of isomeric hydrogen ranges from δ4.7 to 5.3 ppm, suggesting that EPS-LP1 contains both α- and β-glycosidic bonds. The ^13^C NMR spectrum serves as an indicator of the residual amount of polysaccharide within the samples. Furthermore, it allows for the analysis and determination of the number of polysaccharide residues along with their associated configurations by examining the peak numbers corresponding to isomerized carbons exhibiting chemical shifts between δ95 ppm and δ110 ppm. The ^13^C NMR spectrum of EPS-LP1 is illustrated in Figure 3D, where heteromorphic carbon signals were identified at δ102.99, 102.63, 98.71, 99.62, and 98.15 ppm. Notably, the most prominent peak was observed at δ99.62 ppm, which indicates that EPS-LP1 comprises five glycosidic bonds, which is consistent with the methylation analysis. Further detailed information regarding the positioning and sequence of these five glycosidic bonds will be elucidated in future studies.

### 3.6. Antioxidant Activity of EPS-LP1

**Figure 4:**
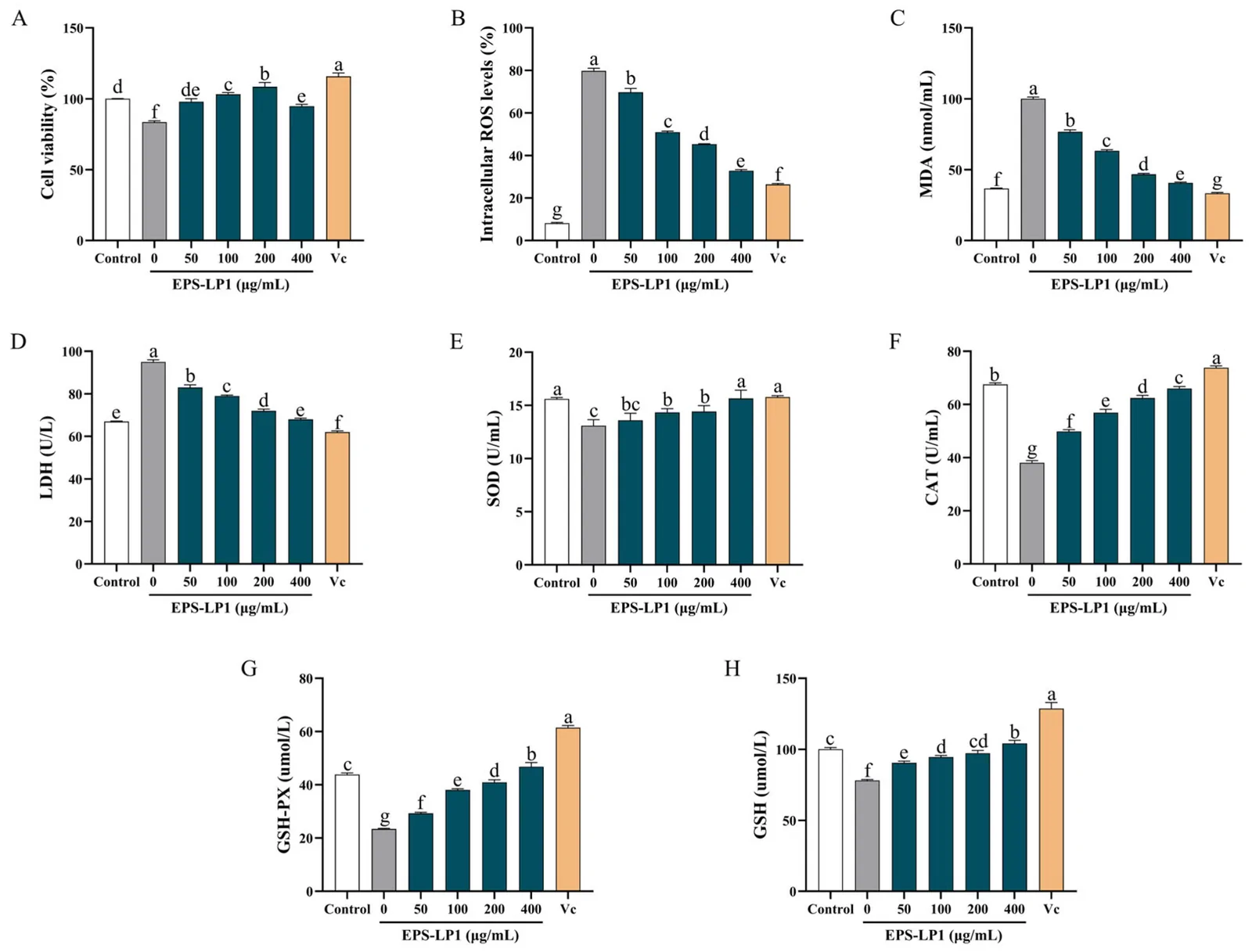
Effects of EPS-LP1 on (**A**) cell viability, the levels of, (**B**) reactive oxygen species (ROS), (**C**) malondialdehyde (MDA), the activities of (**D**) lactate dehydrogenase (LDH), (**E**) superoxide dismutase (SOD), (**F**) catalase (CAT), (**G**) glutathione peroxidase (GSH-Px), and (**H**) GSH in RAW264.7 cells induced by H_2_O_2_. Different letters represent different significance levels from the control group (*p* < 0.05).

As illustrated in Figure 4A, the vitality of RAW264.7 cells induced by H_2_O_2_ was significantly enhanced by the blank control group, negative control group, and EPS-LP1 treatment group. Notably, the EPS-LP1 group exhibited the most pronounced enhancement in cell vitality. This experimental evidence confirms the absence of cytotoxic effects from EPS-LP1 on RAW264.7 cells when administered at concentrations between 50 and 400 µg/mL.

#### 3.6.1. Effect of EPS-LP1 on ROS, MDA, and LDH Generation

To assess the antioxidant properties and underlying mechanisms of EPS-LP1, cellular damage indicators, including reactive oxygen species (ROS), malondialdehyde (MDA), and lactate dehydrogenase (LDH), were quantitatively analyzed. Figure 4A demonstrates that H_2_O_2_-exposed RAW264.7 cells exhibited marked elevations in ROS (Figure 4B), MDA (Figure 4C), and LDH (Figure 4D) concentrations relative to untreated controls, confirming successful induction of oxidative stress. Notably, EPS-LP1 administration substantially attenuated these oxidative markers, with progressive reductions observed in ROS accumulation, lipid peroxidation products (MDA), and lactate dehydrogenase (LDH) across treated groups. The experimental data revealed a concentration-dependent manner of oxidative stress mitigation. Remarkably, at 400 μg/mL EPS-LP1 concentration, ROS, MDA, and LDH concentrations matched those seen in the vitamin C positive control group. This comparative analysis indicated that EPS-LP1 demonstrated significant cytoprotective properties against oxidative cellular damage.

#### 3.6.2. Effects of EPS-LP1 on Enzymatic Antioxidant System in Cells

Generally, SOD, CAT and GSH-Px are enzymes that play critical roles in the antioxidant defense mechanisms within the human body. As illustrated in Figure 4E–G, treatment with varying concentrations of EPS-LP1 significantly enhanced the activities of these intracellular antioxidant enzymes. The findings indicated that EPS-LP1 was capable of partially mitigating oxidative damage to cells induced by H_2_O_2_ (*p* < 0.05). Specifically, at an EPS-LP1 concentration of 400 μg/mL, the measured activities of SOD, GSH-Px and CAT were 15.65 U/mL ± 0.78, 46.83 μmol/L ± 1.56, and 65.98 U/mL ± 0.78, respectively. These results suggest that EPS-LP1 effectively alleviated H_2_O_2_-induced damage in RAW264.7 cells by enhancing the activities of SOD (Figure 4E), CAT (Figure 4F) and GSH-Px (Figure 4G). The antioxidant defense system relies heavily on three crucial enzymes: superoxide dismutase (SOD), catalase (CAT), and glutathione peroxidase (GSH-Px). Experimental results demonstrated that different dosage levels of EPS-LP1 administration markedly boosted the functionality of these cellular antioxidant enzymes, as evidenced in Figure 4E–G. This therapeutic intervention showed statistically significant capacity (*p* < 0.05) to counteract hydrogen peroxide-induced oxidative stress in cellular structures. Notably, when cells were exposed to 400 μg/mL EPS-LP1, the enzymatic activities of SOD, CAT and GSH-Px reached peak performance levels, effectively neutralizing free radicals generated during oxidative damage. The findings indicate that EPS-LP1 demonstrated protective effects against H_2_O_2_-induced oxidative stress through significant enhancement of antioxidant enzyme activities, specifically superoxide dismutase (illustrated in Figure 4E), catalase (shown in Figure 4F), and glutathione peroxidase (detailed in Figure 4G).

#### 3.6.3. Effects of EPS-LP1 on Non-Enzymatic Antioxidant System in Cells

Non-enzymatic antioxidant systems, including glutathione, critically contribute to preserving cellular redox equilibrium. To assess EPS-mediated restoration of oxidative impairment, investigations were conducted to monitor alterations in GSH concentrations within RAW264.7 macrophages. Experimental data from Figure 4H revealed that at 400 μg/mL EPS-LP1 dosage, intracellular GSH levels measured 104.109 μmol/L ± 2.24, showing no significant difference from baseline measurements in untreated cells. These observations indicate that EPS-LP1 administration effectively mitigated oxidative stress-induced GSH reduction while maintaining physiological antioxidant reserves comparable to normal cellular conditions antioxidants while also boosting the cellular non-enzymatic antioxidant defense mechanisms.

### 3.7. Immunomodulatory Activity of EPS-LP1

**Figure 5:**
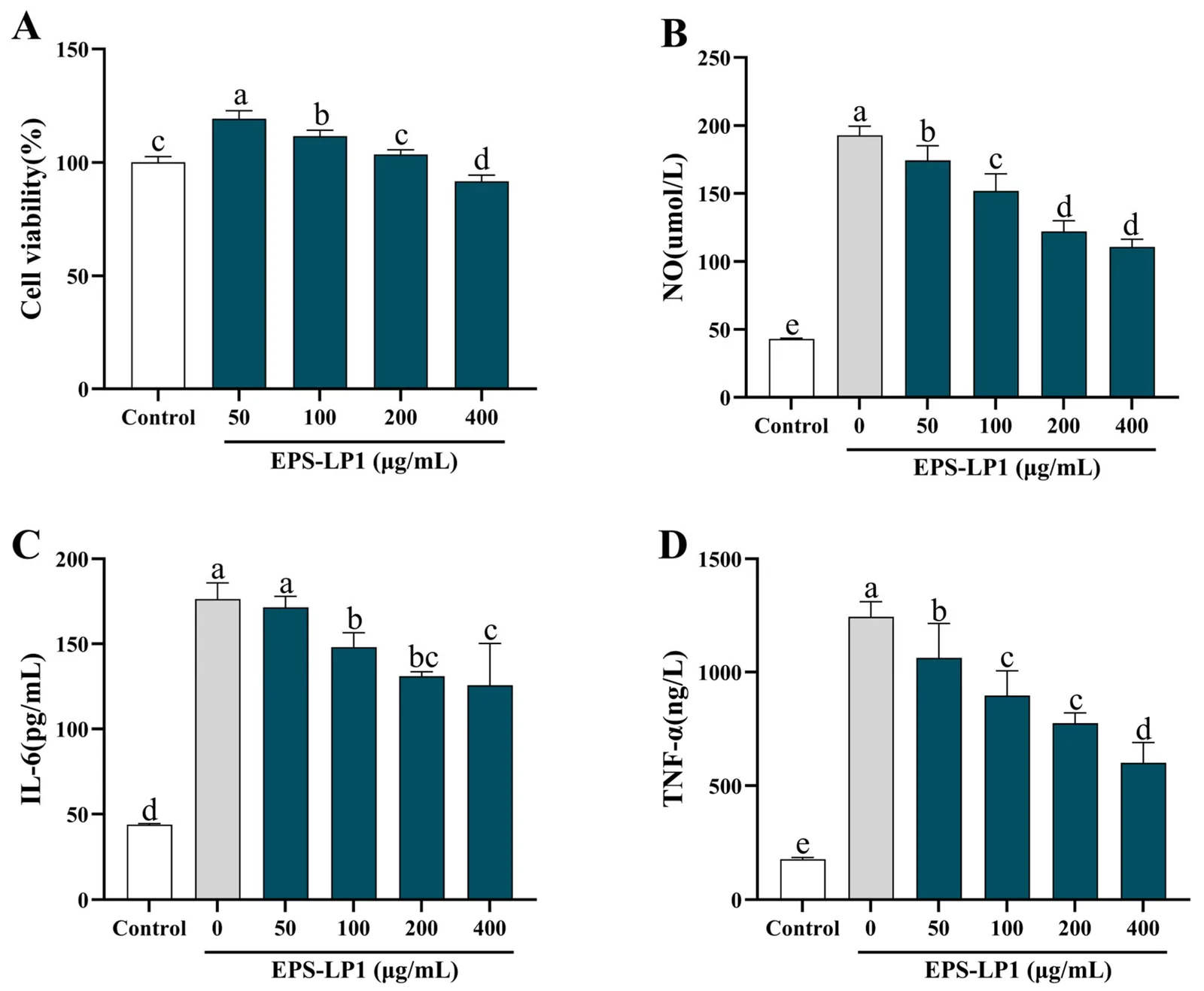
Effects of EPS-LP1 on (**A**) cell viability, (**B**) nitric oxide (NO) production, expression of (**C**) interleukin 6 (IL-6), (**D**) tumor necrosis factor-α (TNF-α) in RAW264.7 cells induced by lipopolysaccharide (LPS). Different letters represent different significance from the control group (*p* < 0.05).

As depicted in Figure 5A, EPS-LP1’s impact on RAW264.7 cell viability under LPS stimulation was examined. Experimental findings revealed comparable cellular viability levels when comparing EPS-LP1 treatments (50–400 μg/mL) with both untreated controls and positive reference groups, showing no statistically meaningful variation. Subsequent immunological evaluations employed five distinct EPS-LP1 concentrations (0, 50, 100, 200, 400 μg/mL) for cellular response assessment, with the 200 μg/mL dosage being specifically selected for subsequent protein expression analysis via western blot methodology.

#### 3.7.1. Effects of EPS-LP1 on NO Production and Cytokines

Immunoregulation is intricately linked to the expression of cytokines, including NO, IL-6, and TNF-α. Figure 5B–D illustrates the secretion levels of these three cytokines in RAW264.7 cells treated with EPS-LP1. The observed decrease in cytokine levels was found to be concentration-dependent on EPS; as the concentration of EPS-LP1 increased, there was a notable decline in cellular pro-inflammatory factors. Furthermore, when compared to the untreated group, high-concentration treatment significantly contributed to reducing NO levels.

#### 3.7.2. Effect of EPS-LP1 on MAPK Signaling Pathway

**Figure 6:**
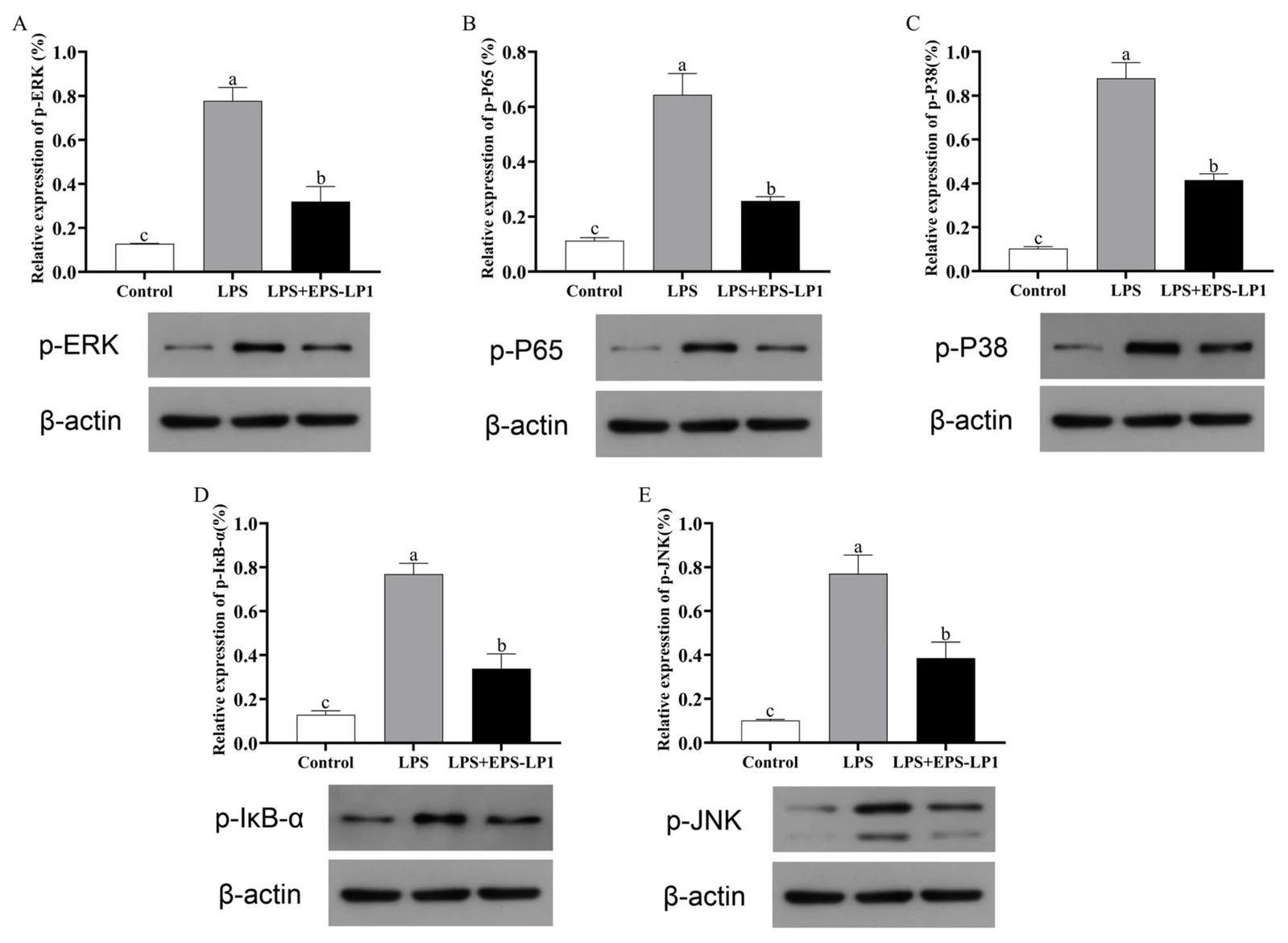
Effects of EPS-LP1 on the MAPK pathway (**A**–**C**) in RAW264.7 cells; effects of EPS-LP1 on the NF-κB pathway (**D**,**E**) in RAW264.7 cells. Different letters represent different significance levels from the control group (*p* < 0.05).

The MAPK signaling cascade features three critical elements (ERK, JNK, and P38 kinases) that regulate immune cell activation. Our investigation focused on EPS-LP1’s influence on the activation states of these kinases. As demonstrated in Figure 6A–C, exposure to LPS markedly increased phosphorylated forms of all three MAPK components (*p* < 0.05), suggesting enhanced immunoregulatory capacity through this pathway. Conversely, administration of EPS-LP1 treatment showed EPS-LP1 treatment resulted in decreased phosphorylation levels of ERK, JNK, and P38 compared to LPS-stimulated conditions, though these levels still remained significantly higher than those in the untreated control group (*p* < 0.05).

#### 3.7.3. Effect of EPS-LP1 on NF-κB Signaling Pathway

NF-κB acts as a pivotal transcriptional controller involved in inflammatory responses and immune regulation processes. Figure 6D,E display the activation states of IκB-α and JNK proteins in LPS-stimulated RAW264.7 macrophages. Experimental data revealed that EPS-LP1 administration markedly suppressed the phosphorylation status of these signaling molecules relative to LPS-induced control groups. This evidence suggests that the therapeutic efficacy of EPS-LP1 might be mediated through interference with critical inflammatory signaling pathways. The potential mechanism underlying EPS-LP1’s immunomodulatory effects in RAW264.7 macrophages could involve interference with NF-κB signaling cascades. Experimental evidence suggests this compound might exert its biological activity through targeted suppression of critical transcriptional regulators in inflammatory pathways.

## 4. Discussion

The results of an in-depth study and analysis of EPS-LP1, a neutral polysaccharide composed of various sugar monomers, were thoroughly examined. The monosaccharide composition of EPS-LP1 includes galactose (Gal), glucose (Glu), and mannose (Man), with corresponding glycosidic bond types identified as 1-Gal*p*, 4-Manp, 1,6-Glc*p*, 1,4-Glc*p*, and 4,6-Gal*p*. These specific configurations of sugar residues and their interconnecting bond patterns play a crucial role in determining the macromolecule’s bioactive properties, particularly influencing its functional capabilities in biological systems.

Mannose-rich polysaccharides exhibit diverse bioactive properties, including oxidative stress mitigation and immune system modulation [38]. Experimental studies revealed that these carbohydrate polymers effectively counteracted hydrogen peroxide-induced cellular damage in murine macrophage cells (RAW264.7), while simultaneously demonstrating potent anti-inflammatory effects. The investigated exopolysaccharide EPS-LP1, characterized by a molecular mass of approximately 55.6 kDa, displayed significant antioxidant capacity and immune-enhancing effects. These effects can potentially be attributed to its unique composition, comprising essential monosaccharides coupled with its elevated molecular weight. Furthermore, EPS-LP1 demonstrated the capability to protect the body against oxidative damage by enhancing enzymatic oxidation activity and elevating antioxidant levels.

Oxidative stress is defined as an imbalance between the production of reactive oxygen species (ROS)/nitrogen species (RNS) and the organism’s capacity to mitigate their effects through antioxidant defense mechanisms. This condition can arise from either an increase in ROS/RNS production or a decrease in the organism’s ability to mount an effective antioxidant response. The phenomenon manifests as reduced effectiveness of intrinsic mechanisms in protecting cellular components from oxidative deterioration, particularly affecting vital biomolecules, including hydrogen peroxide, which interacts with cellular biomolecules such as proteins and nucleic acids present in cellular tissues [39]. When cells are exposed to hydrogen peroxide, they generate substantial amounts of oxidant ROS, which can transform lipid components of cell membranes into lipid peroxides. These lipid peroxides are subsequently degraded into MDA. Concurrently, as cellular damage occurs, a substantial amount of LDH is released from the cells. Following treatment with EPS-LP1, levels of ROS, MDA, and LDH in vitro exhibited a dose-dependent reduction, as illustrated in Figure 4. EPS-LP1 demonstrated its capacity to protect cells from oxidative stress. The body’s antioxidant defense system relies on both enzymatic and non-enzymatic components to eliminate excess ROS. Enzymes such as SOD, CAT, and GSH-Px play crucial roles in maintaining oxidative homeostasis within biological systems [40]. SOD converts superoxide anion radicals into hydrogen peroxide, which is then decomposed into non-toxic substances through the synergistic action of GSH-Px and CAT. The enzymatic activities of GSH-Px and CAT were analyzed alongside glutathione’s role as a crucial non-enzymatic antioxidant in free radical neutralization. To clarify EPS-LP1’s antioxidant action mechanism, comprehensive evaluations were performed on its regulatory effects regarding SOD, GSH-Px, and CAT enzyme activation, coupled with intracellular glutathione concentration measurements. Experimental data indicated that EPS-LP1 substantially increased SOD, GSH-Px, and CAT enzymatic performance, matching the enhancement patterns seen with flavonoid compounds. Moreover, EPS-LP1 exhibits a noteworthy ability to elevate glutathione levels. In summary, EPS-LP1 improved the enzymatic antioxidant capacity of cells while mitigating cellular damage by enhancing the non-enzymatic antioxidant system.

To elucidate the mechanism underlying the immunomodulatory activity of EPS-LP1, we conducted this study. Within the concentration range of 0–400 μg/mL, EPS-LP1 effectively inhibited the production of NO and suppressed the expression of pro-inflammatory cytokines, including TNF-α and IL-6. This investigation focuses on the inflammatory immunoactivity of EPS-LP1, which has been isolated and purified from *L. plantarum*. The NF-κB transcription factor plays a pivotal role in immune signaling and is associated with various human diseases, such as autoimmune disorders, lymphoproliferative conditions, atopic diseases, and inflammatory disorders [41]. To assess the impact of EPS-LP1 on the NF-κB signaling pathway, we measured the expression levels of p-IκB-α and p-P65. Following LPS induction, there was a significant increase in the phosphorylation levels of IκB-α and P65. However, treatment with EPS-LP1 resulted in a marked reduction in these phosphorylation levels, indicating its involvement in modulating the NF-κB signaling pathway. Figure 6 illustrates that EPS-LP1 potentially functions by protecting against the degradation of IκB-α while inhibiting its phosphorylation as well as that of P65.

**Figure 7:**
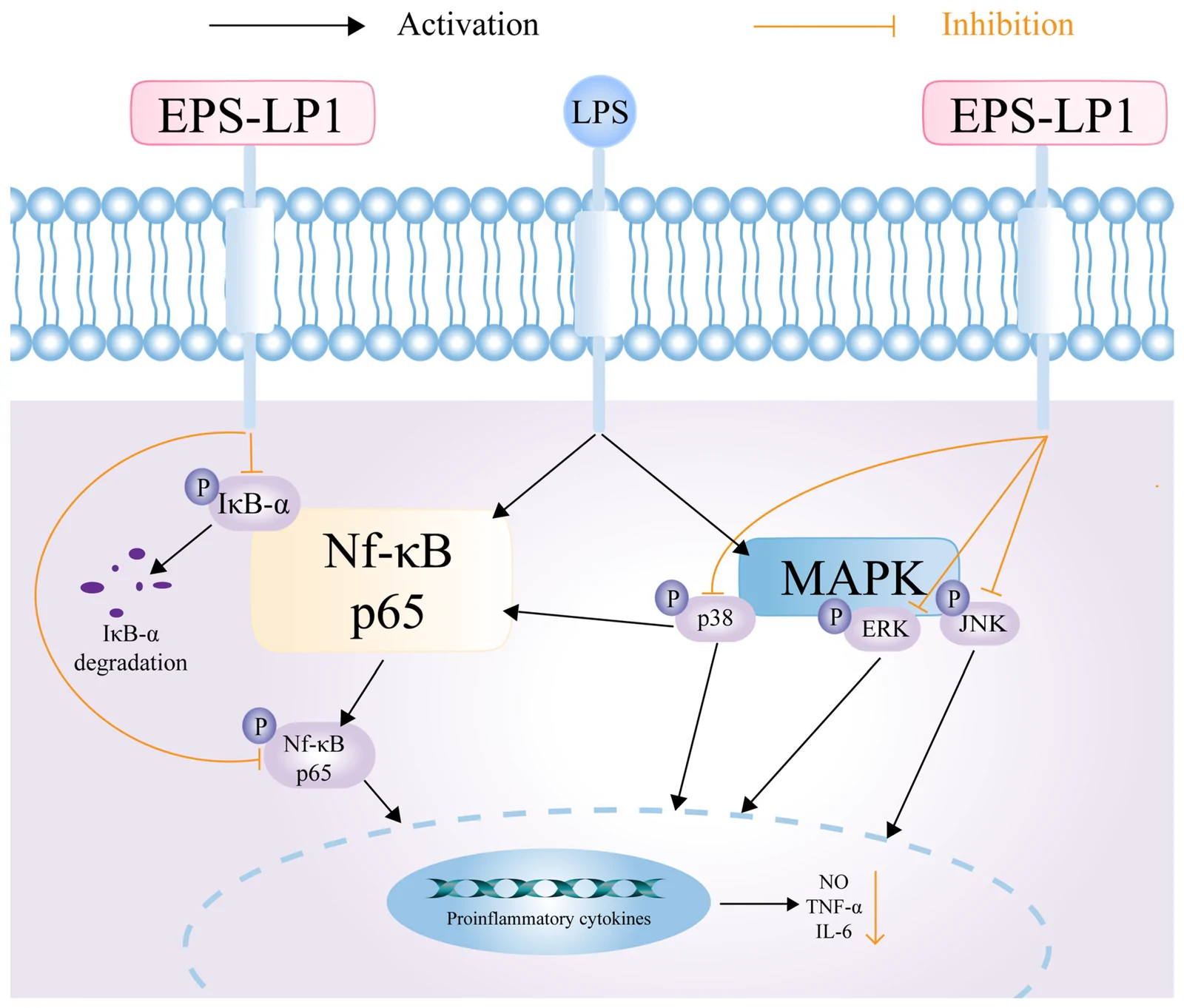
The possible mechanism of EPS-LP1 inhibiting inflammation in RAW264.7 cells.

The MAPK signaling cascade stands as an essential upstream regulator in inflammatory pathogenesis, facilitating the transduction of extracellular stimuli to intracellular compartments while modulating diverse cellular responses [42]. Within this kinase family, ERK, JNK, and P38 emerge as principal mediators of pro-inflammatory signaling networks implicated in various pathological states, including but not limited to oncogenesis, autoimmune dysregulation, chronic inflammatory disorders, and neurological degeneration [43]. The activation of these kinases through phosphorylation constitutes a fundamental mechanism governing cellular responses to inflammatory challenges. The secretion of inflammatory mediators and cytokines triggered by inflammation represents a critical biological process. Our research examined EPS-LP1’s impact on MAPK pathway components, specifically analyzing phosphorylation changes in ERK, JNK, and P38 within LPS-stimulated cellular models. Experimental data revealed that EPS-LP1 administration caused a marked decrease in phosphorylated ERK, JNK, and P38 levels compared to LPS-treated controls (Figure 6). These results indicate that the anti-inflammatory properties of EPS-LP1 may stem from its regulatory action on MAPK signaling pathways. In the presence of EPS-LP1, it can inhibit LPS-induced stress, reduce the phosphorylation levels of ERK, JNK, and P38, regulate the NF-κB and MAPK signaling pathways, thereby leading to a decrease in nitric oxide (NO) and immune cytokine levels (TNF-α and IL-6), and restore normal immune activity (Figure 7).

## 5. Conclusions

The neutral extracellular polysaccharide EPS-LP1 was extracted and purified from *L. plantarum* DMDL 9010. The molecular weight, microscopic morphology, monosaccharide composition, principal functional groups, and NMR spectra of EPS-LP1 were characterized. EPS-LP1 is comprised of glycosidic bonds, including 1-Gal*p*, 4-Man*p*, 1,6-Glc*p*, 1,4-Glc*p*, and 4,6-Gal*p*. It exhibits an smooth and rod like structure along with commendable thermal stability. Exposure to H_2_O_2_-induced oxidative stress demonstrated that EPS-LP1 acts as a catalytic antioxidant capable of boosting enzymatic activity while shielding cellular structures from oxidative harm. The polysaccharide effectively counteracted the deterioration of antioxidant capacity and reinforced cellular defense mechanisms by enhancing non-enzymatic antioxidant components. Experimental data indicated EPS‑LP1 regulates the MAPK pathway by specifically inhibiting phosphorylation events in ERK, JNK, and P38 kinases. Notably, concurrent suppression occurred in the activation markers of inflammatory pathways, with measurable decreases observed in phosphorylated IκB-α and NF-κB (P65) signaling molecules.

Experimental data revealed inhibitory effects observed within the NF-κB signaling cascade, accompanied by a marked reduction in pro-inflammatory cytokine production. These findings collectively suggest that the isolated EPS-LP1 emerges as a novel therapeutic candidate for modulating inflammatory responses through dual mechanisms of pathway suppression and cytokine regulation.

## Data Availability

The original contributions presented in this study are included in the article/Supplementary Material. Further inquiries can be directed to the corresponding author.

## Supplementary Materials

The following supporting information can be downloaded at: https://www.mdpi.com/article/10.3390/nu17142265/s1; Table S1: The chromatographic condition of purification; Table S2: Methylation analyses of reduced EPS-LP1; Table S3: Chromatographic condition of monosaccharides composition. Figure S1: Chromatograms of EPS purified by DEAE-cellulose-52 anion exchange column.
