# Supplementary material for: Identification, Antioxidant and Immunomodulatory Activities of a Neutral Exopolysaccharide from Lactiplantibacillus plantarum DMDL 9010
Source: Nutrients. 2025 Jul 9;17(14):2265. doi: 10.3390/nu17142265 (PMC12298170; doi:10.3390/nu17142265)
Supplement: Supplementary file 1 [file nutrients-17-02265-s001.zip › nutrients-3733181-supplementary.pdf]

Supplementary Tab 1. The chromatographic condition of purification

	DEAE-Cellulose-52 anion exchange column	Sephadex G-75 column
Mobile phase and elution program	Eluted with gradient concentrations of sodium chloride solutions (0, 0.1, 0.3, and 0.5 M)	Eluted with deionized water
Flow rate	1.5 mL/min	0.5 mL/min

Supplementary Tab 2. Methylation analyses of reduced EPS-LP1.

**Linkage type**	**Methylation product**	**Retention time (min)**	**Main MS (m/z)**	**Molar percentage (%)**
t-Gal*p*	1,5-di-*O*-acetyl-2,3,4,6-tetra-*O*-methyl galactitol	9.475	60,71,87,102,129,145,179,205	1.016
t-Man*p*	1,5-di-*O*-acetyl-2,3,4,6-tetra-*O*-methyl mannitol	9.579	60,87,102,118,145,162,205	9.874
6-Glc*p*	1,5,6-tri-*O*-acetyl-2,3,4-tri-*O*-methyl glucitol	14.51	60,87,102,118,162,189,207,233	4.355
4-Glc*p*	1,4,5-tri-*O*-acetyl-2,3,6-tri-*O*-methyl glucitol	14.86	71,87,118,162,233	78.693
4,6-Gal*p*	1,4,5,6-tetra-*O*-acetyl-2,3-di-*O*-methyl galactitol	19.17	57,102,118,149,167,201,261,338	6.062

Supplementary Tab 3. Chromatographic condition of monosaccharides composition.

	Parameters
Model number	ICS5000+, Thermo Fisher Scientific, MA, USA
Mobile phase	A: H_2O; B: 100 mol/L NaOH
Chromatographic column	4.0×250 mm, 10μm
Temperature	30℃
Flow rate	0.5 mL/min
Gradient elution process	0→30.0→30.1→45→45.1→60min
Solvent B	2.5→20→40→40→2.5→2.5%

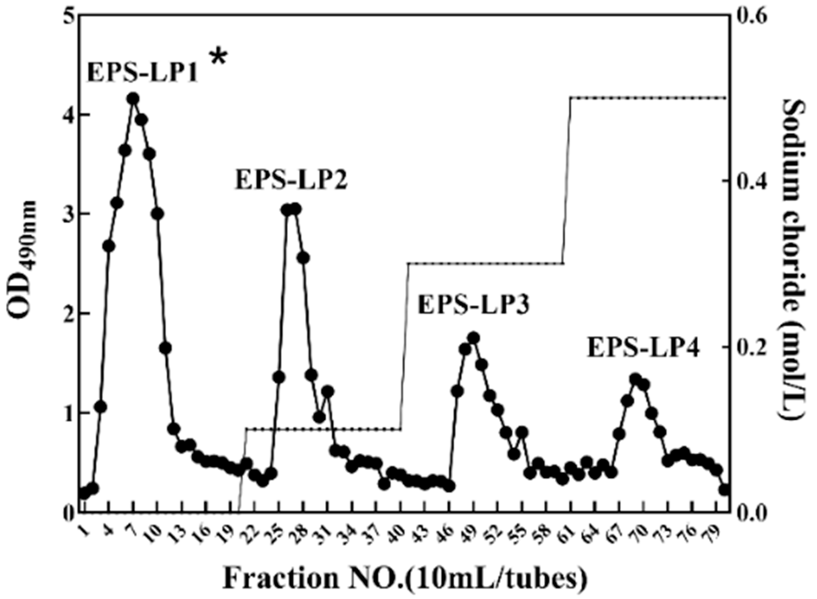

Supplementary Figure 1. Chromatograms of EPS purified by DEAE cellulose-52 anion exchange column
